# A very low thymus function identifies patients with substantial increased risk for long-term mortality after kidney transplantation

**DOI:** 10.1186/s12979-020-00175-z

**Published:** 2020-02-13

**Authors:** Michiel G. H. Betjes, Anton W. Langerak, Mariska Klepper, Nicolle H. R. Litjens

**Affiliations:** 1 Department of Internal Medicine, section Nephrology and Transplantation, Erasmus MC, University Medical Centre, Rotterdam, the Netherlands; 2 Department of Immunology, Erasmus MC, University Medical Centre, Rotterdam, The Netherlands

**Keywords:** Kidney transplantation, Mortality, Premature ageing, Thymus, naïve T cells;recent thymic emigrants

## Abstract

**Background:**

End-stage renal disease is associated with premature ageing of the T cell immune system but inter-individual variation is substantial. The hypothesis was tested that advanced immunological T cell ageing assessed by peripheral T cell differentiation increases the long-term mortality risk after renal transplantation.

**Results:**

Circulating T cells of 211 recipients of a kidney from a living donor were analyzed before and in the first year after transplantation. The number of CD31-positive naive T cells (as a marker for recent thymic emigrants) and the differentiation status of the memory T cells was assessed. Thirty recipients died during follow-up of at least 5 years. Absolute numbers of naive CD4^+^ (living:258 cells/μl vs. deceased:101 cells/μl, *p* < 0.001) and naive CD8^+^ T cells (living:97 cells/μl vs. deceased:37 cells/μl, p < 0.001) were significantly lower in the deceased group prior to transplantation. In a multivariate proportional hazard analysis the number of naive CD4^+^ T cells remained associated with all-cause mortality (HR 0.98, CI 0.98–0.99, *p* < 0.001). The low number of naive T cells in the deceased patient group was primarily caused by a decrease in recent thymic emigrants (i.e. less CD31^+^ naive T cells) indicating a lowered thymus function. In addition, the physiological age-related compensatory increase in CD31^−^ naïve T cells was not observed. Within the first year after transplantation, the number and characteristics of naive T cells remained stable.

**Conclusions:**

A severe reduction in circulating naïve T cells because of a decrease in recent thymic emigrants is highly associated with all-cause mortality after renal transplantation.

## Background

Progressive loss of renal function leading to end-stage renal disease (ESRD) is associated with premature ageing of the T-cell system. The pro-inflammatory environment resulting from loss of renal function [1] is marked by a lower thymic output, increased T-cell differentiation, telomere shortening and skewing of the T cell receptor (TCR) repertoire [2–4].

The changes in the peripheral T-cell compartment of ESRD patients resemble the physiological changes in the immune system of healthy elderly individuals with the immunological age of ESRD patients on average being increased by 15–20 years compared to their chronological T-cell age [4–6].

One of the most consistent and prominent features of ESRD-related premature aging is a lowered thymic output. This is characterized by a decreased number in newly formed naive T cells (i.e. recent thymic emigrants (RTE)) in the circulation. These RTE can either be recognized by the presence of T cell receptor excision circles (circular DNA remnants of the TCR gene rearrangement during thymic maturation) or CD31 expression on naïve CD4 T cells, and close correlation exists between both assays in healthy controls and ESRD patients [4, 7]. As the number of differentiated memory T cells increases with age, both mechanisms together reduce the percentage of naive T cells in the circulation to a relatively high extent [4, 8, 9]. Furthermore, the increased proliferation and differentiation reduces the T-cell telomere length and increases skewing of the TCR repertoire [2, 3].

The driving force behind ESRD-related premature ageing is believed to be the pro-inflammatory environment that arises with retention of uremic waste products, which leads to increased oxidative stress and inflammation [1, 6, 10]. The prematurely aged T cell system of ESRD patients is associated with a reduced vaccination response [11–14] and an increased infection susceptibility [15]. After renal transplantation (RT), increased numbers of highly differentiated T cells are associated with a decreased risk for early acute rejection [16].

Currently, it is not known whether parameters of a prematurely aged T-cell system in ESRD patients are associated with long term survival. In this study, we investigated the association between thymic output measured by CD31 positive naïve CD4 T cells and T-cell differentiation status in recipients prior to renal transplantation with all-cause mortality at follow-up.

## Results

### Recipients characteristics and all-cause mortality

Recipients characteristics are shown in Table 1. The median patient age was 57 years and the majority of the recipients (91%) underwent a renal transplantation for the first time. Thirty recipients (median age 63 year, range 26–78) deceased at a median of 40 months after transplantation (range 6–74 months). The percentage of patients receiving high dose steroids and/or T cell depleting therapy for acute rejection was not significantly different between the living and deceased recipient group (data not shown).

**Table 1:** Clinical and demographical characteristics of renal allograft recipients (*n* = 211) prior to transplantation

Age in years, median (range)	57 (19–79)
Male/female	62–38%
Follow-up time in months, median (range)	74 (0–97)
Living kidney donor, *n* (%)	211 (100%)
Previous kidney transplant, *n* (%)	19 (9.0%)
Pre-emptive transplantation, *n* (%)	91 (43.1%)
Anti-rejection therapy	39 (18.4%)
T cell depleting therapy for rejection	18 (8.5%)
Cardiovascular disease prior to transplantation, *n* (%)	53 (25.1%)
Distribution of underlying kidney disease	
Nephrosclerosis/hypertension	22.7%
Primary glomerulopathies	13.7%
Diabetes mellitus	19.9%
Urinary tract infections/ stones	2.8%
Reflux nephropathy	4.7%
Polycystic kidney disease	19.0%
Other	11.8%
Unknown	5.2%
CMV seropositive*	59%
Recipients deceased during follow-up	30
Cause of death	
cardiovascular disease	20.0%
infection	26.7%
cancer	26.7%
other	3.3%
unknown	23.3%

### Pre-transplant T cell ageing parameters and survival

The average number of different T cell subsets is shown in Table 2.

**Table 2:** Immunological T cell parameters of renal allograft recipients prior to transplantation

	Alive (*n* = 181)^*^	Deceased (*n* = 30)*	*p*-value
Total CD4 T cells	720 (418)**	404 (203)	0.001
Naive CD4 T cells	207 (233)	94 (78)	< 0.001
Naïve CD4 CD31pos T cells	119 (156)	59 (59)	< 0.001
Naive CD4 CD31neg T cells	66 (84)	35 (35)	< 0.001
Memory CD4 T cells	471 (273)	321 (205)	0.018
Central memory CD4 T cells	289 (223)	169 (147)	0.004
Effector memory CD4 T cells	*155 (124)*	117 (153)	0.4
Temra CD4 T cells	10 (17)	7 (11)	0.1
CD28null memory CD4 T cells	16 (53)	25 (69)	0.4
Total CD8 T cells	356 (231)	381 (565)	0.3
Naïve CD8 T cells	67 (91)	27 (35)	< 0.001
Naïve CD8 CD31pos T cells	64 (89)	26 (30)	< 0.001
Naive CD8 CD31neg T cells	2 (2)	1 (3)	0.6
Memory CD8 T cells	256 (208)	299 (571)	0.039
Central memory CD8 T cells	16 (21)	14 (15)	0.7
Effector memory CD8 T cells	109 (94)	125 (214)	0.2
Temra CD8	97 (153)	149 (340)	0.2
CD28null memory CD8 T cells	119 (172)	172 (399)	0.1
CD4/CD8 T cell ratio	2.0 (1.6)	1.2 (1.3)	0.008
CMV seropositive***	57.8%	66.7%	0.6

*Recipients alive and deceased at time of follow-up, **cell numbers are expressed in cells/μL in medians with interquartile range between parenthesis, ***percentage of recipients with detectable serum anti-cytomegalovirus antibodies

**Fig. 1:**
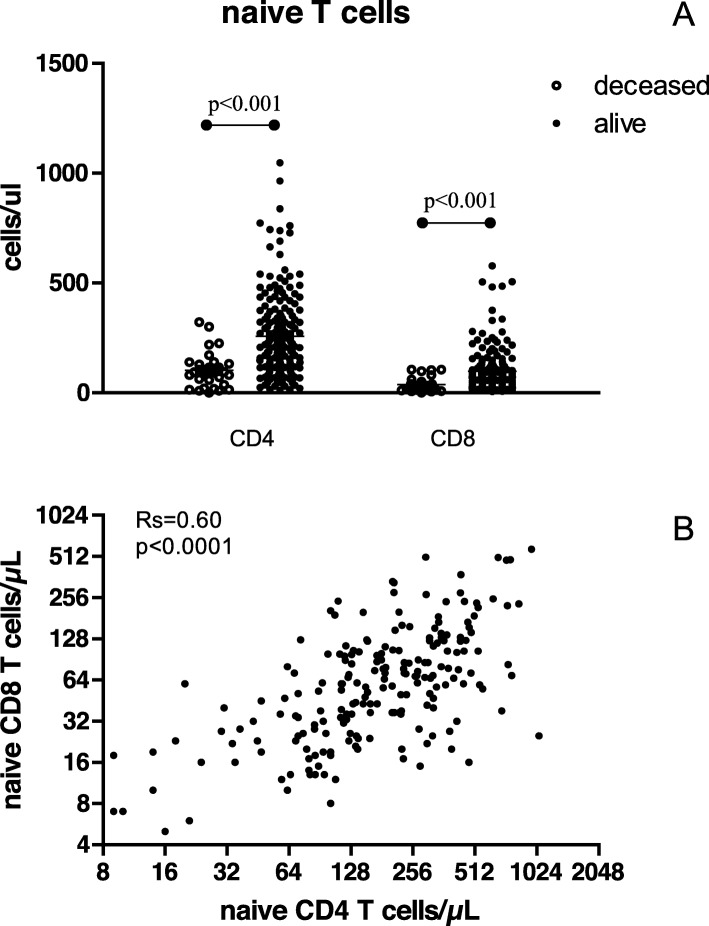
Naïve T cells in relation to recipient survival after transplantation. Recipient’s numbers of naïve CD4^+^ and CD8^+^ T cells at the time of kidney transplantation divided in the recipients alive and deceased at time of follow-up (**a**). In the lower graph the correlation (Rs, Spearman rank correlation coefficient) between naïve CD4^+^ and CD8^+^ T cells numbers is shown (**b**)

The deceased recipient group had a significantly lower total number of circulating CD4^+^ T cells as a result of decreased numbers of naïve CD4^+^ (Fig. 1A) and central-memory T cells. Also within the CD8^+^ T cell population the naïve T cells (Fig. 1A) were significantly decreased, but this was accompanied by an increase in the cell count of total and more differentiated subpopulations of memory T cells (effector memory and Temra subpopulations). As a result, the total number of CD8^+^ T cells was similar between the living and deceased group of recipients. Cell counts of naïve CD4^+^ and CD8^+^ T cells were significantly correlated (Fig. 1B, Rs = 0.56, *p* < 0.001).

**Fig. 2:**
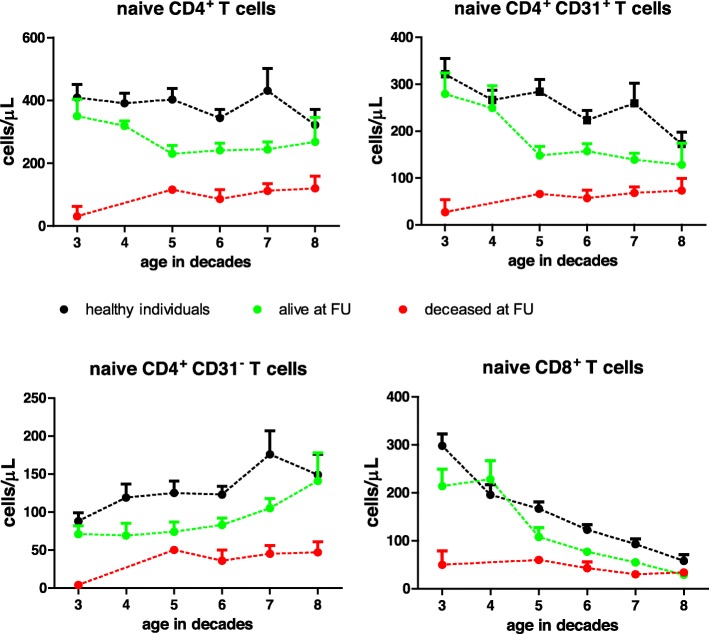
Total number of naïve T cells and CD31 positive and negative CD4^+^ naïve T cells in relation to age. Total naïve CD4^+^, CD31 positive (CD31^+^) and CD31 negative (CD31^−^) naive CD4^+^ T cells and total naïve CD8^+^ T cells are shown for renal transplant recipients (n = 211) and healthy controls (n = 239). The cross-sectional data are represented in means with standard error of the mean for healthy controls (black dots), recipients alive (green dots) and deceased recipients at the time of follow-up (red dots). Age is shown in decades on the X-axis. Dashed interconnecting lines are added to facilitate interpretation of the graph

The number of naïve T cells showed a progressive decreases with age in both healthy individuals and ESRD patients. Confirming data of a previous publication [4], these numbers are on average significantly lowered at all decades in the ESRD patient group leading to a biological age difference of 20 years (Fig. 2). However, at every decade the average number of naïve CD4^+^ and CD8^+^ T cells was lower in the deceased recipient group compared with the living recipient group.

Multivariate proportional hazard logistic regression analysis (Table 4) confirmed that independent of age, a lower number of either CD4^+^ (HR 0.99, 95% CI 0.98–0.99, *p* < 0.0001) or CD8^+^ naïve T cells (HR 0.98, 95% CI 0.96–0.99, *p* < 0.001) was significantly associated with all-cause mortality. When combining both CD4^+^ and CD8^+^ naïve T cell numbers in the model, only the number of naïve CD4^+^ T cells remained significantly associated with all-cause mortality at follow-up (HR 0.99, 95% CI 0.98–0.99, p < 0.001). The interaction term for CD4^+^ and CD8^+^ naïve T cell was not statistically significant (not shown). As expected, similar results were obtained using CD31pos naïve T cells instead of total naïve T cell as a variable in the logistic regression analysis (data not shown).

A positive CMV serostatus is an independent factor associated with a (modest) decreased number of circulating naïve T cells in ESRD patients [17]. This effect was also observed in the current cohort of patients, but independent of CMV serostatus the group alive at follow up had almost twice as much naïve T cells (and CD31pos naïve T cells) as the deceased group (Additional file 1: Table S1). Uni- and multivariate statistical analysis also did not show a significant relation between a CMV positive serostatus and mortality after transplantation (Tables 3 and 4).

**Table 3:** Univariate Cox regression analysis for outcome death after transplantation

	HR	95% CI	p-value
Male/female	0.73	0.35–1.5	0.39
Age at transplantation (per year)	1.05	1.02–1.09	0.004
Pre-emptive transplantation (no dialysis)	0.24	0.09–0.64	0.004
Cardiovascular disease prior to transplantation	3.18	1.55–6.52	0.002
Log C-reactive protein (mg/ml)	2.51	1.26–4.98	0.009
Naive CD4 T cells (per cell/ul)	0.99	0.98–0.99	< 0.0001
Naïve CD8 T cells (per cell/ul)	0.98	0.97–0.99	0.001
CD4/CD8 ratio	0.57	0.38–0.86	0.007
CMV positive serostatus	1.41	0.66–3.10	0.36

**Table 4:** Multivariate Cox regression analysis for outcome death after transplantation

	Hazard ratio	95% CI	p-value
Multivariate model with CD4/CD8 ratio			
Male sex	0.36	0.16–0.78	0.011
Age at transplantation (per year)	1.07	1.03–1.11	<.001
Pre-emptive transplantation (no dialysis)	0.25	0.09–0.70	0.008
Cardiovascular disease prior to transplantation	2.19	0.92–4.45	0.08
Log C-reactive protein (mg/ml)	1.82	0.83–3.97	0.134
CD4/CD8 ratio	0.57	0.37–0.86	0.008
Multivariate model with CMV serostatus			
Male sex	0.40	0.18–0.86	0.02
Age at transplantation (per year)	1.06	1.02–1.1	0.001
Pre-emptive transplantation (no dialysis)	0.26	0.10–0.74	0.010
Cardiovascular disease prior to transplantation	1.76	0.81–3.81	0.152
Log C-reactive protein (mg/ml)	2.11	0.96–4.66	0.064
CMV positive serostatus	1.14	0.53–2.46	0.73
Multivariate model with naïve CD4 T cells			
Male sex	0.29	0.28–0.66	0.036
Age at transplantation (per year)	1.06	1.02–1.09	0.002
Pre-emptive transplantation	0.25	0.09–0.68	0.006
Cardiovascular disease prior to transplantation	1.57	0.70–3.50	0.27
Log C-reactive protein (mg/ml)	1.81	0.83–4.84	0.036
Naive CD4 T cells (per cell/ul)	0.99	0.98–0.99	< 0.001
Multivariate model with naïve CD8 T cells			
Male sex	0.42	0.19–0.92	0.030
Age at transplantation (per year)	1.04	0.99–1.07	0.056
Pre-emptive transplantation	0.26	0.09–0.68	0.005
Cardiovascular disease prior to transplantation	1.61	0.77–3.48	0.22
Log C-reactive protein (mg/ml)	2.09	1.01–4.33	0.115
Naive CD8 T cells (per cell/ul)	0.98	0.96–0.99	0.006

### Naive T cells and the CD4/CD8 ratio

Several studies have shown that an inverted CD4/CD8 T-cell ratio is associated with immunological ageing and associated with poor survival [18, 19]. An important contributor to this inverted ratio is CMV-seropositivity [20, 21]. In our patient group, the CD4/CD8 T-cell ratio was highly associated with all-cause mortality (Tables 2, 3 and 4), primarily as a consequence of a decreased CD4^+^ T cell count in the deceased recipient group compared to the living recipient group. This effect could not be contributed to the influence of CMV-seropositivity as this was equally distributed between both groups (deceased 66.7% vs living 57.8%, *p* = 0.6). In fact, CMV-seropositive recipients on average showed a significantly decreased CD4/CD8 T-cell ratio (CMV^+^ vs. CMV^−^ recipients: 1.85 vs 2.91, *p* < 0.0001). This is because CMV infection leads to a substantial and persisting increase in numbers of circulating CD8^+^ memory T cells [22]. Thus, the decrease in CD4/CD8 T-cell ratio in the deceased recipients group was observed independently of CMV-serostatus (data not shown).

### CD31 expression on naïve T cells and survival

Expression of CD31 on naïve CD4 T cells can be used as a marker for recent thymic emigrants both in healthy individuals and ESRD patients and correlates very closely with results obtained by assaying the T cell receptor excision content [4, 23, 24]. Numbers of CD31^+^ and CD31^−^ naïve CD4^+^ T cells were significantly lower in the deceased recipient group compared to the living recipient group (Table 2). They remained significantly associated with all-cause death including clinical parameters such as age as a variable in the proportional hazard regression analysis (CD31^+^; HR 0.99, 95% CI 0.98–0.99, *p* < 0.001, CD31^−^; HR 0.98, 95% CI 0.97–0.99, *p* = 0.002).

Similar to healthy controls (*n* = 239), the number of CD4^+^ CD31^+^ naïve T cells in the living recipient group declined with age while the number of CD31^−^ naïve T cells increased and (partly) compensated for this decline (Fig. 2). From the age of 70 the average number of CD31^+^ and CD31^−^ contributed roughly equally to the composition of the circulating naïve CD4^+^ T cells.

In contrast, in the deceased recipient group this age-dependent dynamics was not observed and the number of CD31^+^ and CD31^−^ naïve T cells were low in all age decades (Fig. 2).

The number of naïve CD8^+^T cells decreased sharply with age both in healthy individuals and the living recipient group. In contrast to the CD4^+^ naïve T cells, virtually all naïve CD8^+^ T cells express CD31^+^ (see Table 2) which is a known phenomenon and limits its use as a marker for recent thymic emigrants for CD8^+^ T cells [25].

Numbers of naïve CD4^+^ and CD8^+^ T cells did not correlate with any of the clinical and demographical characteristics as shown in Table 1, except for age.

### Dynamics of naïve T cell numbers during the first year after transplantation

**Fig. 3:**
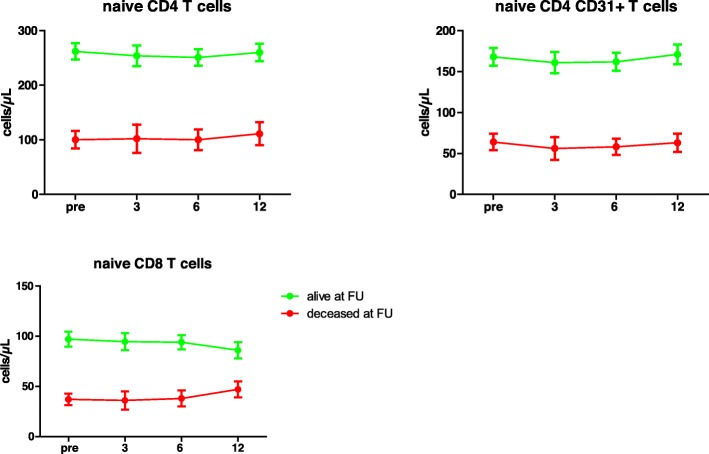
Dynamics of naïve T cells post-transplantation. The longitudinal course of total CD4^+^ (A) and CD8^+^ (C) naïve T cell numbers and the CD31-expressing subset of naïve CD4^+^ T cells after renal transplantation are shown. On the x-axis the time of sampling is shown: before transplantation (0) and the months after transplantation (3, 6, 12). Cell numbers are given in means with standard error of the mean forrecipients alive (green dots) and deceased recipients at the time of follow-up (red dots)

In both the deceased and living recipient group the numbers of naïve CD4 and CD8 T cells remained stable during the first year after transplantation (Fig. 3). Also, longitudinal follow-up during the first year after transplantation did not show significant changes in the number of CD31^+^ and CD31^−^ CD4^+^ naïve T cells in both the deceased and living recipient group (Fig. 3).

## Discussion

In this prospective study the hypothesis was tested that the degree of premature immunological aging at the time of renal transplantation is associated with long-term all-cause mortality thereafter. Of all immunological parameters tested, the number of circulating naïve T cells was significantly associated with all-cause mortality at a follow-up of 5–7 years after transplantation. In particular, the number of CD31^+^ CD4^+^ naïve T cells was substantially lower in every age group in the deceased recipient group, without a compensatory increase in CD31^−^ CD4^+^ naïve T cells.

CD31^+^ naïve T cells are considered to be equivalent to naïve T cells that have recently emigrated from the thymus and/or have not undergone prior TCR-induced proliferation. Although maybe not the perfect parameter, the number of CD31^+^ naïve T cells could be considered as an indicator of thymus function [23, 25]. With increasing age the thymus involutes resulting in an almost linear decrease in CD31^+^ CD4^+^ naïve T cells [4, 26]. Specifically, the circulating naïve CD8^+^ T cell subset shrinks substantially [27] while the number of CD31^−^ CD4^+^ naïve T cells shows a compensatory increase [28]. This normal physiological ageing process is also observed in patients with chronic renal failure but their thymic output of naïve T cells, either measured by CD31 expression or content of TCR excision circles (TCR) [7, 26], shows premature biological ageing by 15–20 years. This premature ageing of the thymus function was confirmed in this study and recipients most affected by immunological ageing, showing a severe decreased thymic output, appeared to have the highest mortality rate after transplantation. Currently, it is not known why some patients have such a low thymic output but low levels of interleukin 7 in ESRD patients may play a role [29]. A previous study showed that the decline in naïve T cell numbers is associated with the progression of stage of renal insufficiency, indicative of a causative role for eGFR loss [29]. It is well-documented that patients with ESRD have a pro-inflammatory immune status [30]. Related to this phenomenon, activation-induced apoptosis of naïve T cells is higher in ESRD patients [4]. A shift from lymphoid to myeloid progenitor stem cells in the bone marrow, induced by inflammation-associated epigenetic changes, offers another possible explanation [1]. However, currently there are no studies that have tested these hypotheses in an experimental model.

The inter-individual variation of thymic output and decline over time is substantial both in ESRD patients and healthy individuals and ageing is the most prominent factor influencing this process. A positive CMV serostatus is associated with a (modest) decrease in numbers of naïve T cells but was not a confounding factor in this study.

The observation of a lack in compensatory increase of CD31^−^ naïve T cells in the deceased recipient group is intriguing but cannot be readily explained. In previous studies, an increased susceptibility for activation-induced cell death was identified as possible contributor to naïve T cell lymphopenia in ESRD patients [4, 9]. Although speculative, increased activation-induced cell death may be more prominently present in the deceased recipient group, e.g. because of a greater degree of systemic inflammation. As a general marker of systemic inflammation the serum C-reactive protein concentrations were measured at time of T cell analysis but showed no relation with naïve T cell numbers (data not shown). However, in a recent study by Chiu et al. a significant correlation was observed between naïve CD8 T cell numbers and serum CRP level [8]. In that study virtually all patients were CMV seropositive which may indicate that CMV, by causing increased systemic inflammation [31, 32], could induce naïve T cell depletion.

In ESRD patients, the reintroduction of adequate renal function by successful renal transplantation leads to a rapid normalization of uremia-associated inflammation and oxidative stress [33, 34]. These processes are thought to underlie the impact of renal failure on the immune system but their reversal does not lead to a change of T cell ageing parameters [35]. However, we could not show a relation between early post-transplantation infection risk and e.g. low numbers of naïve T cells [36]. In the current study, the thymic output in both the deceased and living group of recipients remained remarkably stable, indicating that even the most severely affected patients do not even partially restore their thymic function.

In a previous study, it was shown that the number of circulating naïve T cells closely correlates with the content of naïve T cells in the lymph nodes [37]. Therefore, redistribution of naïve T cells to the lymphoid tissue does not seem likely. However, it has recently become clear that naïve T cells may be compartmentalized early in life and can reside in many non-lymphoid tissues [38, 39]. Whether all these naïve T cell populations in tissues are similarly affected by renal failure is not known.

The low thymic output may be directly related to an increased mortality, e.g. by a decreased immune response to new pathogens, or may act as a sensitive biomarker for an aged biological (immune) system. This is a highly relevant question as when the latter assumption holds true, rejuvenation of thymic function alone may not be sufficient to decrease mortality risk.

The number of naïve T cells in elderly individuals has been linked to frailty and mortality [40, 41] although this was not found in all studies [42]. With regard to recipients of a renal transplantation low numbers of CD4^+^ T cells are associated with cardiovascular mortality [43] and opportunistic infection with Pneumocystis jiroveci pneumonia after transplantation [44]. In the latter study, it was noted that the lymphopenia was a relatively stable feature of the patients involved, confirming our observation that ESRD-related lymphopenia is not affected by renal transplantation. The current data, in accordance with previous studies, shows that ESRD-related lymphopenia is largely due to a loss of circulating naïve T cells. Therefore, the results of the above mentioned studies are most likely explained by the naïve CD4^+^ T cell lymphopenia which is underlying the total CD4^+^ T cell lymphopenia. In addition, the lower CD4^+^ T cell count in the deceased recipients groups lowers the CD4/CD8 T-cell ratio which is therefore significantly associated with all-cause mortality. This is in accordance with the results of previous studies in healthy adults [45]. CMV-seropositivity is a potential strong confounder of this association because of the CMV-infection associated increase in the CD8 T cells count [22] leading to a significantly decreased CD4/CD8 T-cell ratio. As the frequency of CMV seropositivity increases with age and is negatively related to socio-economic status, the CD4/CD8 T-cell ratio as biomarker for mortality risk is highly susceptible for misinterpretation [30].

There are some obvious limitations to the current study that are worth mentioning. Our cohort of kidney transplant recipients all received a kidney of a living donor. This lead to a very homogenous cohort of recipients, not receiving T cell depletion therapy, almost no delayed graft function and excellent one-year patient and graft survival. In other transplantation programs, the majority of kidneys are from deceased donors and T cell depletion is regularly given as induction therapy. Therefore, the generalizability of the current findings is unknown at present. Another limitation is the relative low numbers of events (recipients deceased at follow up) limiting extensive multivariate analysis and allowing for confounders not identified.

## Conclusions

A severely lowered number of circulating naïve T cells, indicative of a very low thymic output, before renal transplantation is not reversed by renal transplantation per se and is strongly associated with long term all-cause mortality.

## Methods

### Study population

All ESRD patients who received a kidney from a living-donor in the period from November 1st 2010 to October 1st 2013 were considered for participation. Follow-up of patients included in the study was until November 2018.

All patients received a similar immunosuppressive regimen. This included induction therapy with basiliximab (Simulect®, Novartis) and maintenance therapy with tacrolimus (Prograf®, Astellas Pharma), mycophenolate mofetil (Cellcept®, Roche), and glucocorticoids. Clinical variables were assessed as shown in Table 1, including age, gender, CMV-seropositivity, number of prior renal transplantations, underlying cause of renal failure, pre-emptive RT. The primary end-point of the study was all-cause mortality. Cause of mortality was documented as either cardiovascular disease, cancer, infectious disease, other and unknown. HLA-typing was performed according to the international standards (American Society for Histocompatibility and Immunogenetics/the European Federation for Immunogenetics) using serologic and DNA-based techniques. PRAs were determined at the laboratory of the blood bank in Leiden, the Netherlands.

### PBMC isolation

By using Ficoll-Paque Plus (GE healthcare, Uppsala, Sweden), peripheral blood mononuclear cells (PBMC) were isolated from heparinized blood samples. Blood was drawn from RT-recipients the day before RT, and at 3, 6 and 12 months after RT. Isolated PBMCs were stored at -150°C with a minimum amount of 10 × 10^6^ cells per vial.

### T cell differentiation status and absolute numbers of T cell subsets

To determine the T-cell differentiation status a whole blood staining was performed based upon a study by Sallusto et al. [46] as described in detail previously [4]. Naïve T cells were defined by the expression on either CD4 or CD8, CCR7 and the absence of CD45RO. Absolute numbers of the different T cell subsets were determined via a Trucount staining as described by Bouvy et al. [47].

### Statistics

The difference between continuous variables was assessed with the Mann–Whitney U test. Normal distribution of data was assessed by the Shapiro-Wilk test of normality. Not-normal distributed data were represented in medians with interquartile range. The difference between categorical variables was analyzed either with the Pearson’s chi-squared test or with the Fisher’s exact test depending on the expected values in any of the cells of a contingency table. Cox proportional hazard analysis was used to assess the association between immunological parameters and the outcome all-cause death, including relevant clinical parameters (age, sex, pre-emptive transplantation, cardiovascular disease, C-reactive protein) in the model. Univariate Cox proportional hazards analysis was used to identify clinical and demographic variables associated with death at follow-up. Variables with a *p*-value of ≤ 0.1 and sexe were considered for further analysis by multivariate logistic regression to calculate hazard ratios and corresponding confidence intervals. PH assumption of variables were tested by visual inspection of log-minuslog graphs and further tested by assessment of time-dependency using the Cox regression with time-dependent covariate module in SPSS. All variables met the demands of PH unless stated otherwise. Interaction terms that met statistical significance (*p* < 0.05) were included in the multivariate model. Correlation matrices were made for the assessment of significant correlations. Absence of collinearity in the model covariates was formally assessed by calculating the variance inflation factor.

The significance level (p-value) was two-tailed and an α of 0.05 was used for all analyses. Statistical analyses were performed using SPSS® version 21.0 for Windows® (SPSS Inc., IL, USA) and GraphPad Prism 5 (CA, USA). Figures were created with GraphPad Prism 5 (CA, USA).

## Supplementary information

**Additional file 1: Table S1.** Naïve T cell numbers before transplantation in recipients stratified for CMV serostatus and survival after transplantation.

## Data Availability

All data generated or analysed during this study are included in this published article.

## Abbreviations

CMV: Cytomegalovirus
ESRD: End-stage renal disease
RT: Renal transplantation
RTE: Recent thymic emigrant
TCR: T cell receptor
