# Supplementary material for: A very low thymus function identifies patients with substantial increased risk for long-term mortality after kidney transplantation
Source: Immun Ageing. 2020 Feb 13;17:4. doi: 10.1186/s12979-020-00175-z (PMC7020578; doi:10.1186/s12979-020-00175-z)
Supplement: Supplementary file 1 — Additional file 1: Table S1. Naïve T cell numbers before transplantation in recipients stratified for CMV serostatus and survival after transplantation. [file 12979_2020_175_MOESM1_ESM.docx]

| Table S1. Naïve T cell numbers before transplantation in recipients stratified for CMV serostatus and survival after transplantation | | |
| --- | --- | --- |
|  |  |  |
| All recipients | CMV seropositive (n=124) | CMV seronegative (n=86) |
|  |  |  |
| Naive CD4 T cells * | 129 (187)^$^ | 233 (237) |
| Naive CD4 CD31pos T cells | 90 (124)^$^ | 153 (149) |
| Naïve CD8 T cells | 51 (73)^$^ | 74 (93) |
| Naïve CD8 CD31pos T cells | 44 (68)^$^ | 74 (91) |
|  |  |  |
| Recipients alive at follow up | CMV seropositive (n=104) | CMV seronegative (n=76) |
|  |  |  |
| Naive CD4 T cells | 165 (193)^#^ | 286 (261^##^ |
| Naive CD4 CD31pos T cells | 103 (140)^#^ | 167 (140)^##^ |
| Naïve CD8 T cells | 59 (73)^#^ | 77 (97)^##^ |
| Naïve CD8 CD31pos T cells | 56 (72)^#^ | 75 (95)^##^ |
|  |  |  |
| Recipient deceased at follow up | CMV seropositive (n=20) | CMV seronegative (n=10) |
|  |  |  |
| Naive CD4 T cells | 88 (57) | 135 (129) |
| Naive CD4 CD31pos T cells | 55 (46) | 76 (52) |
| Naïve CD8 T cells | 21 (27) | 42 (63) |
| Naïve CD8 CD31pos T cells | 20 (25) | 39 (60) |
|  |  |  |
| Data represent number of cells per ul prior to transplantation given in medians with interquartile range between parenthesis. ^$^p-value <0.01 for comparing CMV seropositive to CMV seronegative recipients. ^#^p-value <0.001 for comparing CMV seropositive recipients alive and deceased at follow up. ^##^ p-value <0.001 for comparing CMV seronegative recipients alive and deceased at follow up. | | |
